# Forearm Flexor Muscles in Children with Cerebral Palsy Are Weak, Thin and Stiff

**DOI:** 10.3389/fncom.2017.00030

**Published:** 2017-04-25

**Authors:** Ferdinand von Walden, Kian Jalaleddini, Björn Evertsson, Johanna Friberg, Francisco J. Valero-Cuevas, Eva Pontén

**Affiliations:** ^1^Department of Women's and Children's Health, Karolinska InstituteStockholm, Sweden; ^2^Division of Biokinesiology and Physical Therapy, University of Southern CaliforniaLos Angeles, CA, USA; ^3^Department of Neurology, Karolinska Hospital HuddingeStockholm, Sweden; ^4^Karolinska InstituteStockholm, Sweden; ^5^Department of Biomedical Engineering, University of Southern CaliforniaLos Angeles, CA, USA; ^6^Department of Pediatric orthopedic Surgery, Astrid Lindgren's Children HospitalStockholm, Sweden

**Keywords:** cerebral palsy, skeletal muscle, muscle stiffness, muscle size, upper limb

## Abstract

Children with cerebral palsy (CP) often develop reduced passive range of motion with age. The determining factor underlying this process is believed to be progressive development of contracture in skeletal muscle that likely changes the biomechanics of the joints. Consequently, to identify the underlying mechanisms, we modeled the mechanical characteristics of the forearm flexors acting across the wrist joint. We investigated skeletal muscle strength (Grippit®) and passive stiffness and viscosity of the forearm flexors in 15 typically developing (TD) children (10 boys/5 girls, mean age 12 years, range 8–18 yrs) and nine children with CP Nine children (6 boys/3 girls, mean age 11 ± 3 years (yrs), range 7–15 yrs) using the NeuroFlexor® apparatus. The muscle stiffness we estimate and report is the instantaneous mechanical response of the tissue that is independent of reflex activity. Furthermore, we assessed cross-sectional area of the flexor carpi radialis (FCR) muscle using ultrasound. Age and body weight did not differ significantly between the two groups. Children with CP had a significantly weaker (−65%, *p* < 0.01) grip and had smaller cross-sectional area (−43%, *p* < 0.01) of the FCR muscle. Passive stiffness of the forearm muscles in children with CP was increased 2-fold (*p* < 0.05) whereas viscosity did not differ significantly between CP and TD children. FCR cross-sectional area correlated to age (*R*^2^ = 0.58, *p* < 0.01), body weight (*R*^2^ = 0.92, *p* < 0.0001) and grip strength (*R*^2^ = 0.82, *p* < 0.0001) in TD children but only to grip strength (*R*^2^ = 0.60, *p* < 0.05) in children with CP. We conclude that children with CP have weaker, thinner, and stiffer forearm flexors as compared to typically developing children.

## Introduction

An insult to the immature, developing brain before the age of two results in a condition clinically referred to as cerebral palsy (CP), characterized by motor impairment (Rosenbaum et al., 2007). Despite that the brain injury is non-progressive, motor function commonly deteriorates over time and a progressive contracture formation, i.e., a decrease in passive range of motion (pROM), is common (Hagglund and Wagner, 2011). For many children, surgical treatment is needed to restore and preserve musculoskeletal function. Clinical experience suggests that contracture formation is due to shortening/stiffening of the musculotendinous complex, as the range of motion of the joint often is practically normal when tendons of contracted muscles are cut during surgery. What causes the increased stiffness and shortness of the muscle is not known, but clinically we know that it affects joint biomechanics.

Children with CP generally have less skeletal muscle mass as compared to typically developing (TD) children (Barrett and Lichtwark, 2010). Recent reports have highlighted stunted growth in the lower limb as a contributing factor to contracture development (Gough and Shortland, 2012) and slowed growth rate has been detected as early as 15-months of age (Herskind et al., 2015). Several investigators have described increased passive stiffness of the calf muscle in children with CP and different tests have shown that the calf muscle is 22–120% stiffer in CP children compared to TD (Ross et al., 2011; de Gooijer-van de Groep et al., 2013; Geertsen et al., 2015). The process starts early and increased whole muscle passive stiffness in the calf has been described as early as 3 years of age (Willerslev-Olsen et al., 2013). Biochemical studies of muscle in CP have shown an increased content of intramuscular collagen (Booth et al., 2001), with an increased amount of connective tissue around fiber bundles i.e., a thickening of the perimysial extracellular matrix (de Bruin et al., 2014). The perimysium is considered to be in a physical continuum with the tendon (Passerieux et al., 2007). Thus, marked collagen deposition in the perimysium could potentially offer an increased resistance to passive stretch.

Less information is available on how skeletal muscle size correlates to joint biomechanics and muscle function for the upper limb. This is unfortunate as the clinical importance of accurate information on skeletal muscle function and biomechanical properties prior to for example tendon transfer surgery of the wrist is of great importance and a guide in clinical decision making. Therefore, we aimed to model the mechanical characteristics of the forearm flexors acting across the wrist joint in children with CP during passive stretch. Second, we aimed to correlate skeletal muscle size and biomechanics to age, body weight and strength.

## Materials and methods

### Participants

Nine children [6 boys/3 girls, mean age 11 ± 3 years (yrs), range 7–15 yrs] with CP (3 bilateral/6 unilateral) scheduled for upper limb surgery at Karolinska University Hospital, Stockholm, Sweden were consecutively included in the study. Inclusion criteria were confirmed CP diagnosis, age between 7 and 18 yrs, and cognitive ability to follow instructions. The children with CP were classified according to the Gross Motor Function Classification Scale (GMFCS) and the Manual ability classification system (MACS). GMFCS I means that the child can walk indoors and outdoors without limitations, and children in GMFCS V has no means of independent mobility. The GMFCS classification has been shown to be the best predictor of treatment results in CP (Shore et al., 2012). Eight of the children were classified as GMFCS I and one child as GMFCS III. The GMFCS III child needed a walking aid indoors and a wheel chair outdoors. MACS I means that the child handles objects easily and successfully with minimal limitation in speed and accuracy, and children in MACS V do not handle objects and has limited ability to perform even simple actions (requires total assistance). The children in the current study were classified as follows; MACS I three children, MACS II four children and MACS III two children.

As controls, a convenience sample (*n* = 15) of TD children was recruited (10 boys/5 girls, mean age 12 yrs, range 8–18 yrs). This study was carried out in accordance with the recommendations of the Regional Ethical Review Board in Stockholm with written informed consent obtained from all subjects and minimum one parent per subject in accordance with the Declaration of Helsinki. The protocol was approved by the Regional Ethical Review Board in Stockholm.

### Clinical assessment and strength measurements

Passive range of motion (pROM) of the wrist was assessed by a physiotherapist or trained medical student using a goniometer. All children were thereafter tested for maximal grip strength using a dedicated device, Grippit® (AB Detektor, Göteborg, Sweden). The arm of the subject was placed in a neutral position (thumb upwards) with the ulnar side resting in a padded semicircular plastic tube. All subjects received verbal encouragement and were instructed to grip the handle as hard as they could and maintain the same intensity for a 10 s period. The handle size was adjustable and each child was allowed to independently choose the handle size.

### Neuroflexor measurements and data processing

For wrist stiffness measurements, we used the ***NeuroFlexor***® (NeuroFlexor® Scientific, Release 0.0.6, Aggero MedTech AB, Solna, Sweden). With the elbow in 90°, the hand was placed on the NeuroFlexor® platform, so that the axes of rotation of the wrist and the platform were aligned. Subjects were instructed to remain relaxed throughout the experiments and the device applied ramp-and-hold perturbations with a velocity of 5°/s, by extending the wrist from 20° palmar flexion to 30° extension. It recorded the angle of the wrist and the forces exerted by the limb and device. For each subject, we recorded 5 trials. We also recorded a trial following removal of the subject's hand from the device (no-hand trial) to account for forces generated by the mechanical properties of the apparatus only.

**Figure 1:**
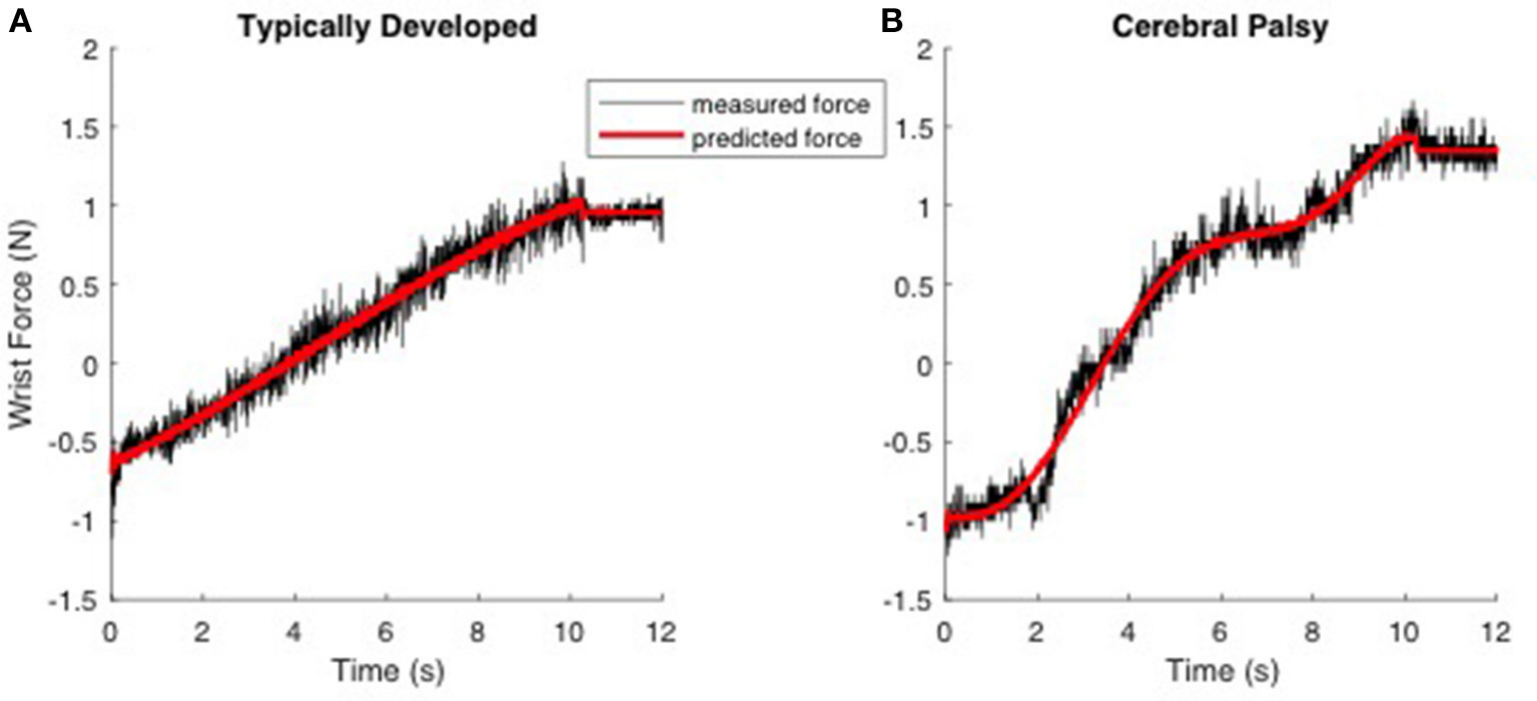
**Typical trials demonstrating the prediction of the measured force using the biomechanical model in response to ramp-and-hold perturbation of 50 degrees with a low velocity (5°/s) experiment**. Left; **(A)** TD. Right; **(B)** CP. The hand is moving from time 0 to 10 s. Data was recorded for another 2 s (time 10–12 s). The black and red line represents measured and predicted forces, respectively.

We subtracted the no-hand force from the with-hand forces and used a linear least-squares technique to identify the biomechanical model (Schouten et al., 2008; Meskers et al., 2015):

$$\begin{array}{l} F_{WH}(t)-F_{NH}(t)=ml^{2}\theta^{\prime \prime }(t)+B\theta^{\prime }(t)+K\theta (t)\;+K_{1}\theta^{2}(t)\\ \;+\;K_{2}\theta^{3}(t)+mgcos(\theta (t)) \end{array}$$

where *F*_*WH*_(*t*) and *F*_*NH*_(*t*) are the recorded forces in the with-hand and no-hand trials, *m* is the mass of the hand (point mass), *l* is the length of the hand from the wrist joint, θ(*t*) is the recorded wrist angle and θ′(*t*), θ″(*t*) are its first and second derivatives (angular velocity and acceleration) computed numerically by differentiating the recorded joint angle, *g* is the gravitational acceleration and finally *K* and *B* are the joint stiffness and viscous parameters, respectively. The second and third-order power of joint angles (θ^2^(*t*), θ^3^(*t*)) were also included in the model to account for nonlinear changes of joint biomechanics as a function of joint angle (Sobhani Tehrani et al., 2013). The main assumption of the model is that the neural component of the force is negligible, which is a fair assumption as the velocity of perturbation was low enough to avoid evoking reflex responses (Jalaleddini et al., 2016). Thus, the muscle stiffness we estimate and report is the instantaneous mechanical response of the tissue that is independent of reflex activity. From this model, we quantified and reported the mean and standard deviation of the stiffness and viscous parameters across the trials. Figure 1 shows typical measured and predicted forces using the biomechanical model. The biomechanical model accounted for 95.5 ± 3.6% of the variance in measured force, on average.

### Skeletal muscle ultra sound assessment and image processing

The cross sectional area of the *flexor carpi radialis muscle* (FCR) was measured with B-mode ultrasonographic equipment with a linear probe (BK medical Flex focus 1202, 8670, scanning frequency 12 MHz Herlev, Denmark). The subjects were instructed to place their elbow on a height adjustable table with the dorsum of the hand toward the examiner and the fingers extended. If this position was difficult for the child to maintain, the caretaker was instructed to assist and hold the arm in the above-described position. Then, the distance from the olecranon to the styloid processes of the ulna was determined using a measuring tape. Skin markings were made at the transition between the proximal and middle third of the forearm, using a black permanent marker, to ensure correct placement of the ultrasound probe. During the ultrasound examination the subject was sitting in a standardized position with the dorsum of the forearm resting flat on a height adjustable table, the elbow at approximately 60° of flexion, arm supinated and fingers extended. Following generous application of water-soluble gel (Gurò Medigel, Gurò s.a.s.—Catenanuova (En)—Italy) the head of the ultrasound probe was placed proximal and parallel to the line marking, and at a right angle to the radius and ulna. The interfaces between subcutaneous adipose tissue and muscle tissue, between muscle tissue and fascia and between muscle tissue and bone were identified on the ultrasonic image. The border of the muscle was outlined by free hand, and the supplied software in the ultrasonograph was used to calculate the cross-sectional area.

### Statistical analysis

Values are reported as means ± SD. Differences in age, body weight, isometric grip strength, skeletal muscle cross sectional area, muscle passive stiffness and viscosity were investigated by the Mann-Whitney-Wilcoxon test. Linear regression was used to investigate correlations between FCR area and age, body weight, grip strength and passive stiffness and viscosity, respectively. Significance level was set at *p* < 0.05 for all statistical comparisons.

## Results

**Figure 2:**
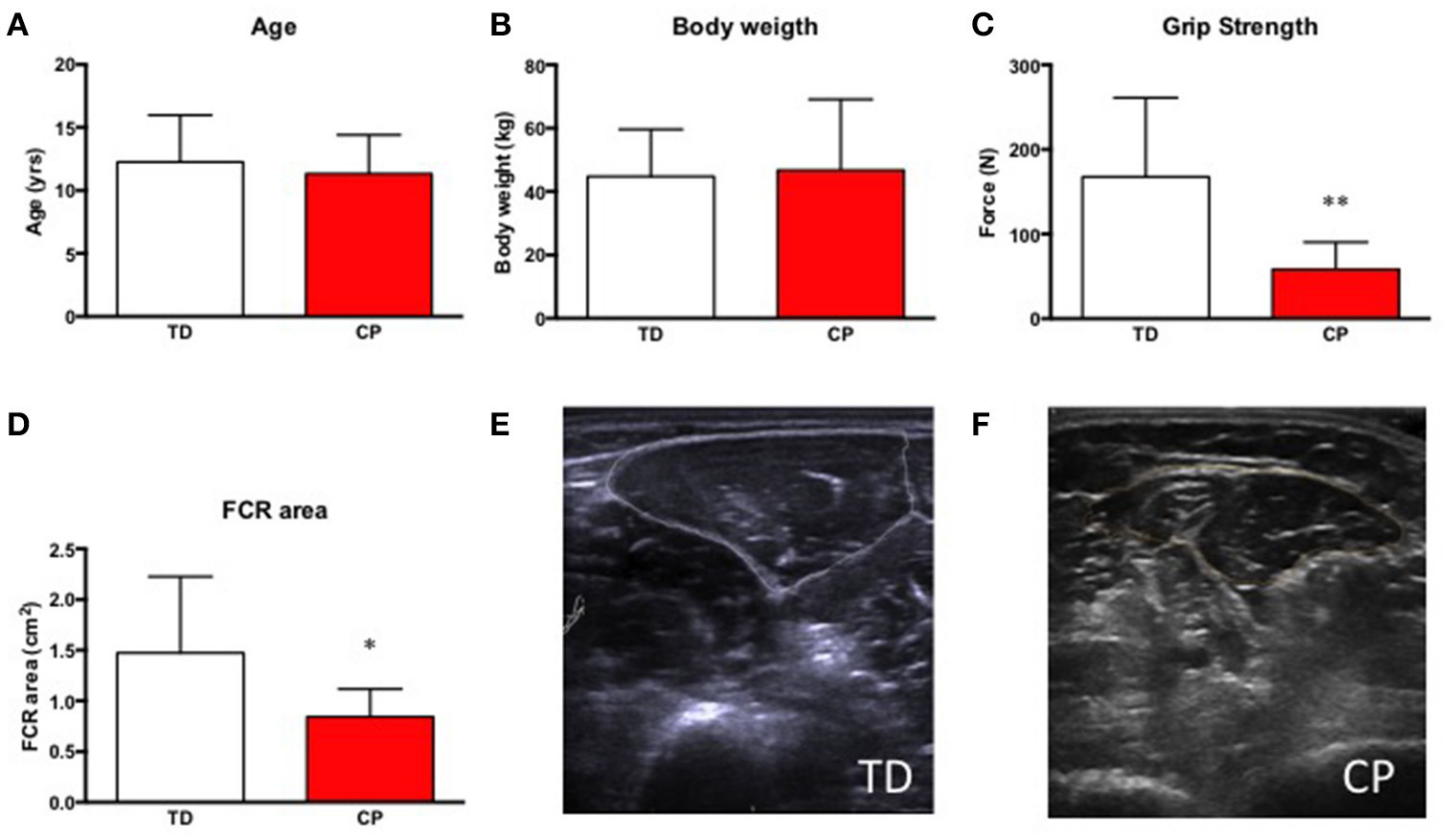
**(A)** Age in years of included research subjects, typically developed children (TD) and children with cerebral palsy (CP). **(B)** Body weight in kg in TD and CP. **(C)** Grip strength (N) in TD and CP. ^**^Denotes significantly different from TD, *p* < 0.01. **(D)** Cross-sectional area of the flexor carpi radialis muscle (FCR) in cm^2^ in TD and CP. ^*^Denotes significantly different from TD, *p* < 0.05. For all bar graphs, TD (white) & CP (red). **(E,F)** Representative ultrasound images of forearm muscles in TD **(E)** and CP **(F)** children. Encircled areas represent FCR.

No difference was seen between the groups with respect to age (CP 11.3 yrs ± 3.1 vs. TD 12.3 yrs ± 3.7, ns) and body weight (CP 46.8 ± 26.1 kg vs. TD 44.7 ± 14.9 kg, ns). FCR cross sectional area was 43% smaller in children with CP as compared to TD children (CP 0.84 cm^2^ ± 0.38 vs. TD 1.47 cm^2^ ± 0.75, *p* < 0.01). As expected, grip strength (10 s isometric contraction) was also significantly lower, −65% in CP as compared to TD children (CP 58.3N ± 32.1 vs. TD 167.5N ± 93.5, *p* < 0.01, Figure 2).

**Figure 3:**
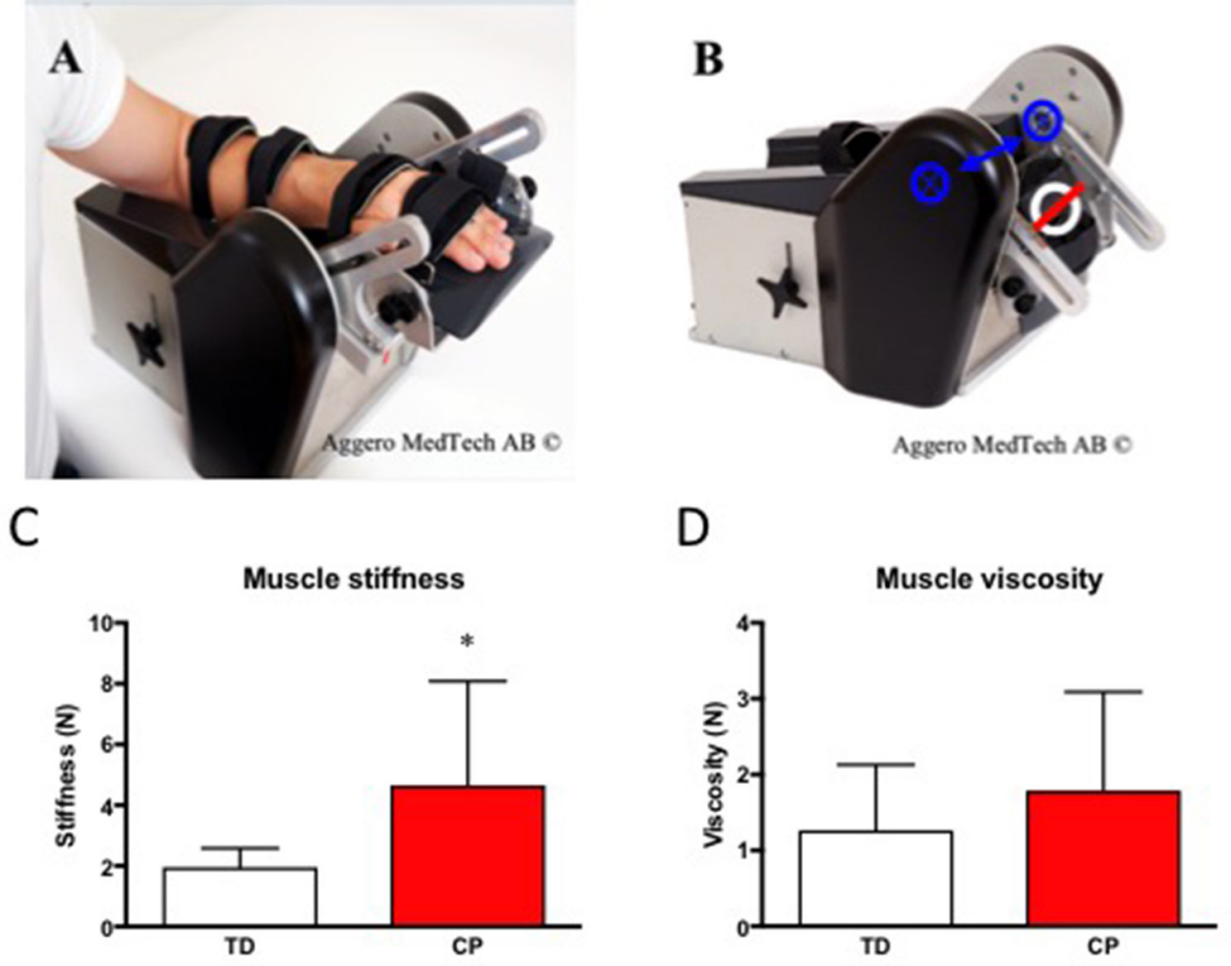
**(A)** The NeuroFlexor® with hand at 30° extension. **(B)** The NeuroFlexor® at 20° Flexion. Blue arrow and crossed rings highlight the axis of rotation. White ring highlights the position of the force sensor. The red line was used to localize the center of the force sensor. **(C)** Skeletal muscle passive stiffness (N) in TD and CP. ^*^Denotes significantly different from TD, *p* < 0.05. **(D)** Skeletal muscle viscosity (N) in TD and CP. For all bar graphs, TD (white) & CP (red). Photos in **(A,B)** were used with permission from Aggero Medtech AB.

Skeletal muscle passive stiffness was increased 2-fold in CP as compared to TD (CP 4.65 ± 3.42 vs. TD 1.96 ± 0.62, *p* < 0.05) whereas skeletal muscle viscosity didn't differ significantly between the groups (CP 1.71 ± 1.22 vs. 1.27 ± 0.86, Figure 3).

**Figure 4:**
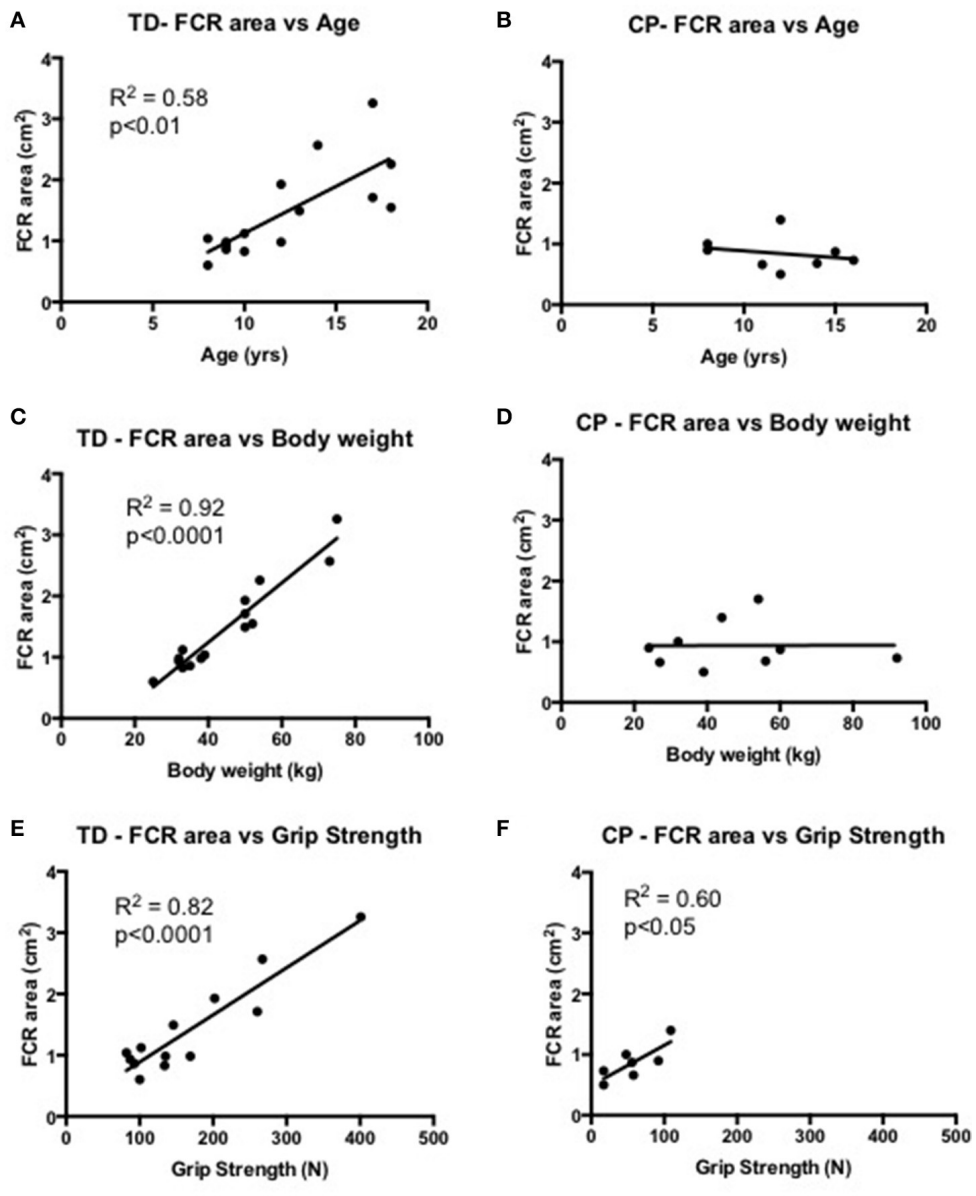
**(A,B)** Correlational analysis of the *flexor carpi radialis muscle* (FCR) cross-sectional area (cm^2^) and age (yrs) in TD (graph **A**) and CP (graph **B**). **(C,D)** Correlational analysis of FCR cross-sectional area (cm^2^) and body weight (kg) in TD (graph **C**) and CP (graph **D**). **(E,F)** Correlational analysis of FCR cross-sectional area (cm^2^) and grip strength (N) in TD (graph **E**) and CP (graph **F**). *R*^2^ and significance level indicated in each graph when relevant.

Skeletal muscle size correlated to age (*R*^2^ = 0.58, *p* < 0.01), body weight (*R*^2^ = 0.92, *p* < 0.0001) and strength (*R*^2^ = 0.58, *p* < 0.01) in TD children. Interestingly, the same relationship between skeletal muscle size did not correlate to age and body weight in children with CP, however still showing a significant correlation to strength (*R*^2^ = 0.60, *p* < 0.05). Skeletal muscle size did not correlate to passive stiffness or viscosity in CP or in TD children (Figure 4).

## Discussion

This study shows that forearm flexor muscles in children with CP are stiffer as compared to TD children. Similar findings have been described for the lower limb, specifically the ankle flexors. Previous reports have estimated the passive tension to be 22–120% higher in CP for the ankle flexor muscles (Ross et al., 2011; de Gooijer-van de Groep et al., 2013; Geertsen et al., 2015). Thus, our finding of a 2-fold increase in skeletal muscle stiffness of the forearm flexors is comparable to data on weight bearing muscles of the lower limb. Information on the current status of the muscle is of great clinical importance when planning treatments, such as stretching, use of splints and surgical interventions e.g., tendon transfer surgery. Tendon lengthening, rerouting and transfer in the upper limb aim to balance the muscles of the wrist, so that the wrist can be maintained in a functional neutral position. All these surgical techniques have a component of dosing—how tight should the tendon transfer be, how long the tendon lengthening? This dosing is highly dependent on how stiff the transferred or lengthened muscle is. Long-term results are difficult to predict, as there often is a progression of contracture formation and stiffness. Abnormalities such as contracture and stiffness in even a few muscles will add yet another obstacle to smooth and efficient movements (Keenan et al., 2009; Kutch and Valero-Cuevas, 2011; Valero-Cuevas et al., 2015) which can progress very quickly in these children.

Spastic CP is characterized by a hyperexcitibility of the stretch reflex (Lance, 1980). Due to the fact that many children with spastic CP eventually develop skeletal muscle contractures, spasticity has been believed to be causative and clinical practice has up until recently been focused on spasticity reducing therapies for the preservation of motor function. However, recent reports have clearly demonstrated that despite good control of spasticity and reduction of muscle tone, by intramuscular botulinum toxin injections (chemical denervation) or selective dorsal rhizotomy that disrupts the reflex loop, skeletal muscle contracture formation progresses (Alhusaini et al., 2011; Tedroff et al., 2014). The poor effect of spasticity reduction on contracture development has raised the question of whether other factors such as growth disturbances and/or alterations in muscle composition are of greater mechanistic importance. Furthermore, recent findings suggest an overestimation of the contribution of spasticity to increased passive tension in muscle of young children with CP and at the same time an underestimation of the presence of contractures (Willerslev-Olsen et al., 2013; Herskind et al., 2015). In a large population study of children with CP, spasticity has been shown to peak around 4 years of age and thereafter decreases and level out at 12 years of age, whereas reductions in passive range of motion i.e., skeletal muscle contractures is progressive (Hagglund and Wagner, 2011). Therefore, we have chosen to focus on the instantaneous mechanical response of the muscle that is independent of reflex activity.

Muscle fiber size increases from 10–12 μm^2^ at birth to 40–60 μm^2^ after puberty (Oertel, 1988). Lexell et al have shown that skeletal muscle fiber size continuously increases with age up until and shortly after puberty. The same study also found a close relationship between fiber size and muscle cross sectional area (Lexell et al., 1992). Children with CP have reduced growth in general (Day et al., 2007). Skeletal muscle growth rate is reduced, evident as early as at 15-months of age (Herskind et al., 2015) and without showing any tendency to catch up later in life (Malaiya et al., 2007; Barber et al., 2011, 2016; Noble et al., 2014b). Just as for passive stiffness of skeletal muscles, deficit in skeletal muscle size in children with CP has so far only been described for the lower limb. The cross sectional area of the FCR muscle was significantly smaller in children with CP as compared to TD children. We have shown in previous studies that wrist flexors in children with CP have a greater fiber size variability and altered myosin composition compared to TD children (Ponten and Stal, 2007). In addition, the sarcomere, the smallest contractile unit of the muscle, has altered properties in CP (Ponten et al., 2007). By using laser diffraction methodology, we have measured sarcomere lengths of wrist flexors intra-operatively during tendon transfer surgeries. With the wrist held in neutral position, the sarcomeres were longer in CP compared to control, and sarcomeres were also longer—i.e., more stretched out- the worse the wrist contracture was (Ponten et al., 2007). This means that the wrist extension movement by the NeuroFlexor® likely results in extreme stress of the sarcomeres, with less overlap of the actin and myosin filaments and more stress on the perimyseal collagen surrounding the fiber bundles (de Bruin et al., 2014). In our study population the cross sectional area of the wrist flexor FCR, as determined by ultrasound, was significantly smaller in CP as compared to TD children and did not correlate to age or body weight. This implies that the differing brain damage the children with CP have, has a greater impact on muscle growth than age and body size. This further supports the impression of great heterogeneity within CP previously reported (Handsfield et al., 2016) and underscores the need for structured, individualized longitudinal follow-up.

Increased skeletal muscle echo intensity as measured by ultrasound has previously been used to infer altered skeletal muscle composition (Heckmatt et al., 1982; Nielsen et al., 2006). Pitcher et al. recently investigated the gastrocnemius muscle of 40 children with cerebral palsy and compared their findings to typically developed age matched controls (*n* = 12) (Pitcher et al., 2015). They show increased skeletal muscle echo intensity in CP and suggest that the underlying cause is due to increased content of non-contractile tissue e.g., collagen. Biochemical analysis of hydroxyproline content, a measure of intramuscular collagen content, correlates to spasticity severity (muscle tone as assessed by Modified Ashworth Scale, MAS) in children with CP (Booth et al., 2001). Staining muscle collagen using Sirius red has in CP shown increased amount of perimyseal collagen surrounding muscle fiber bundles (de Bruin et al., 2014). Similarity, MRI assessment of the lower limb indicates that young adults with CP have higher intramuscular fat content as compared to healthy controls (Noble et al., 2014a). Thus, it's without a doubt that skeletal muscle in individuals with CP with time contains an increased amount of non-contractile material. We interpret this as an indicator of the progressive remodeling in CP muscle. Despite that CP is defined as a non-progressive disease, skeletal muscle pathophysiology seems to continuously worsen over time.

We conclude that children with CP have weaker, thinner and stiffer forearm flexors as compared to typically developed children.

## Author contributions

FvW: Performed experiments, analyzed data, drafted manuscript (MS), designed figures, finalized MS, approved final version of MS. KJ: Analyzed data, drafted MS, designed figures, finalized MS, approved final version of MS. BE: Performed experiments, designed figures, finalized MS, approved final version of MS. JF: Performed experiments, finalized MS, approved final version of MS. FJVC: Conception of study, analyzed data, approved final version of MS. EP: Conception of study, drafted MS, finalized MS, approved final version of MS.

## Funding

The research reported herein was supported by grants from Sunnerdahls handikapp Stiftelse, Norrbacka-Eugeniastiftelsen and Stiftelsen Samariten to FvW; Linnea and Josef Carlssons Stiftelse, Stiftelsen Promobilia and by grants provided by the Stockholm County Council (ALF project) to EP. This material is in part based upon work supported by NIH Grant R01-052345, NIH Grant R01-050520 to FJVC. This project is also supported by the Postdoctoral Research Scholarship from Fonds de Recherche du Québec- Nature et Technologies to KJ.

### Conflict of interest statement

The authors declare that the research was conducted in the absence of any commercial or financial relationships that could be construed as a potential conflict of interest.
